# Semi‐Organic Artificial Photosynthetic System with Engineered Phenoxazinone Derivatives for Photocatalytic Hydrogen Production with Broadened Near‐Infrared Light Harvesting

**DOI:** 10.1002/advs.202501037

**Published:** 2025-02-22

**Authors:** Xiaowen Ruan, Depeng Meng, Minghua Xu, Guozhen Fang, Chunsheng Ding, Jing Leng, Xuan Wang, Kaikai Ba, Haiyan Zhang, Wei Zhang, Tengfeng Xie, Zhifeng Jiang, Jianan Dai, Xiaoqiang Cui, Sai Kishore Ravi

**Affiliations:** ^1^ School of Energy and Envionment City University of Hong Kong Tat Chee Avenue, Kowloon Hong Kong SAR 999077 P. R. China; ^2^ National Key Laboratory of Automotive Chassis Integration and Bionics School of Materials Science and Engineering Key Laboratory of Automobile Materials of MOE Jilin Provincial International Cooperation Key Laboratory of High‐Efficiency Clean Energy Materials Electron Microscopy Center Jilin University 2699 Qianjin Street Changchun 130012 P. R. China; ^3^ State Key Laboratory of Molecular Reaction Dynamics Dalian Institute of Chemical Physics Chinese Academy of Sciences Dalian 116023 P. R. China; ^4^ College of Information Technology Jilin Normal University Siping 136000 P. R. China; ^5^ College of Chemistry Jilin University 2699 Qianjin Street Changchun 130012 P. R. China; ^6^ Institute for Energy Research Jiangsu University Zhenjiang 212013 P. R. China

**Keywords:** artificial photosynthesis, heterojunction, photocatalyst, semi‐organic, solar hydrogen production

## Abstract

Natural photosynthetic systems utilize complex pigment‐protein assemblies for light harvesting across a broad spectral range from UV to near‐infrared, enabling efficient photogeneration and charge separation. Conventional photocatalysts, however, primarily absorb UV (<380 nm) and visible light (380–780 nm), resulting in suboptimal spectral utilization. This study introduces a semi‐organic artificial photosynthetic system that integrates molecularly engineered phenoxazinone derivatives with H‐doped rutile TiO_2_ (H‐TiO_2_) nanorods. Bis(Triphenylamine)Phenoxazinone (BTP) features a phenoxazinone core with two triphenylamine donor groups, enabling light absorption up to 800 nm. Modifying BTP with an additional malononitrile group (MBTP) extends absorption into the NIR region up to 1200 nm. Optimized semi‐organic catalysts with MBTP nanobelts and H‐TiO_2_ nanorods showed an excellent photocatalytic hydrogen evolution rate of 29.4 mmol g^−1^ h^−1^ and 60.4 µmol g^−1^ h^−1^ under UV–vis and NIR irradiation, respectively. Femtosecond transient absorption (fs‐TA) spectroscopy showed rapid electron injection from the photoexcited phenoxazinone derivatives to the H‐TiO_2_ conduction band, indicating efficient charge carrier dynamics. Photoelectrochemical measurements confirmed improved charge transport and reduced recombination in the MBTP‐based system, attributed to the stronger internal electric field and increased dipole moment from the malononitrile modification. These findings highlight the potential of tailored semi‐organic systems for high‐efficiency solar‐to‐hydrogen conversion.

## Graphical abstract

This study introduces a semi‐organic artificial photosynthetic system that integrates molecularly engineered phenoxazinone derivatives with H‐doped rutile TiO_2_ (H‐TiO_2_) nanorods. Optimized semi‐organic catalysts with MBTP nanobelts and H‐TiO_2_ nanorods show an excellent photocatalytic hydrogen evolution rate of 29.4 mmol g^−1^ h^−1^ and 60.4 µmol g^−1^ h^−1^ under UV–vis and NIR irradiation, respectively.

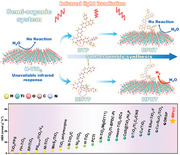

## 1 Introduction

Natural photosynthetic systems achieve efficient solar energy conversion by utilizing a range of pigments, such as chlorophylls and accessory pigments, which are finely tuned to absorb light across a broad spectral range, including ultraviolet (UV, λ< 380 nm), visible (380–780 nm), and into the near‐infrared (NIR, 780–2500 nm) wavelengths.^[^ ^1^, ^2^ ^]^ While chlorophylls primarily absorb light in the blue and red regions, some specialized organisms, like cyanobacteria and algae, employ pigments such as phycobiliproteins and bacteriochlorophylls that extend light absorption into the NIR region.^[^ ^3^, ^4^ ^]^ These pigments capture lower‐energy NIR photons, allowing these organisms to thrive in low‐light environments, such as deep water, where NIR light penetration is more significant. Although these natural pigments do not reach absorption into the deeper NIR regions beyond 900 nm, they exemplify the importance of broad‐spectrum absorption for maximizing solar energy capture and facilitating efficient photogeneration and charge separation.

Artificial photosynthetic systems aim to harness solar energy to drive chemical reactions such as hydrogen production and carbon dioxide reduction, offering a sustainable pathway to renewable fuels.^[^ ^5^, ^6^, ^7^ ^]^ However, many of these systems rely on conventional inorganic photocatalysts, which are typically limited to absorbing ultraviolet (UV) and visible (vis) light, which account for only a portion of the solar spectrum.^[^ ^8^, ^9^, ^10^ ^]^ Specifically, UV light constitutes ≈5% and visible light ≈45%, leaving the near‐infrared (NIR) region, which makes up ≈50% of the solar spectrum, largely unexploited.^[^ ^11^, ^12^ ^]^ NIR light harvesting has found increasing applications in areas such as photodynamic therapy,^[^ ^13^ ^]^ pro‐drug activation,^[^ ^14^ ^]^ and in vivo imaging.^[^ ^15^ ^]^ Despite its potential, most artificial photosynthetic systems are unable to efficiently utilize NIR light due to the narrow absorption range of conventional inorganic photocatalysts and the lower photonic energy of NIR light. This limitation restricts the overall efficiency of these systems.^[^ ^16^ ^]^ Overcoming this limitation is crucial for advancing artificial photosynthetic systems and improving their efficiency across a broader range of applications including hydrogen production, CO_2_ reduction, and beyond.^[^ ^17^ ^]^

To address the challenge of limited light absorption, especially in the NIR region, many researchers have turned to modifying traditional photocatalysts, with titanium dioxide (TiO_2_) being one of the most extensively studied materials. TiO_2_ is valued for its high photostability, low cost, and non‐toxicity, making it a widely used photocatalyst in artificial photosynthetic systems.^[^ ^18^, ^19^, ^20^ ^]^ However, TiO_2_ has a wide bandgap of ≈3.2 eV, which restricts its light absorption primarily to the UV region. In addition to its limited absorption range, TiO_2_ also suffers from high recombination rates of photogenerated charge carriers, which further reduces its efficiency in driving photocatalytic reactions. Developing catalyst design strategies for achieving broadband light absorption along with efficient charge separation and transport is therefore critical.^[^ ^21^, ^22^ ^]^ To enhance the light absorption capabilities of TiO_2_, various strategies have been explored, including the construction of heterojunctions by coupling TiO_2_ with visible light‐active materials such as metal sulfides, plasmonic nanoparticles, and other semiconductors.^[^ ^23^, ^24^ ^]^ While these modifications have successfully extended light absorption into the visible region, the efficient utilization of red (>600 nm) and NIR (>780 nm) light remains a significant challenge.

A promising strategy to overcome the limitations of conventional inorganic photocatalysts is the development of semi‐organic artificial photosynthetic systems. Semi‐artificial photosynthetic systems that integrate pigment‐proteins with materials of complementary absorption characteristics have been explored in our previous works to broaden the spectral coverage of light harvesting across different bio‐photoelectrochemical configurations.^[^ ^25^, ^26^, ^27^ ^]^ Similarly, for photocatalytic hydrogen production, integrating synthetic organic molecules with inorganic semiconductors can extend the light absorption range while still benefiting from the stability and charge transport properties of inorganic photocatalysts. Organic compounds, particularly donor‐acceptor (D‐A) types, have garnered increased attention due to their structural versatility, cost‐effectiveness, and broad light absorption capabilities.^[^ ^28^, ^29^ ^]^ Several specific D‐A type compounds have been investigated for their ability to achieve efficient photocatalytic hydrogen evolution.^[^ ^30^, ^31^ ^]^ For example, Cooper et al.^[^ ^32^ ^]^ have demonstrated that a D‐A type organic compound, 2,6‐bis(4‐cyanophenyl)‐4‐(9‐phenyl‐9H‐carbazol‐3‐yl)pyridine‐3,5‐dicarbonitrile, can alter photocatalytic selectivity through nanomorphology and solid‐state packing. Zhong et al.^[^ ^33^ ^]^ have reported a new D‐A type organic compound, 1,3,6,8‐tetra(di(p‐pyrid‐4‐ylphenyl)amino)pyrene, which acts as a single‐chromophore photocatalyst with superior activity for H_2_ production from water. Despite the significant progress achieved with D‐A compounds in the field of photocatalysis, their application in NIR light harvesting has been largely overlooked, with most research focusing on compounds that absorb only in the UV–vis range.^[^ ^34^ ^]^ Given the unique tunability of D‐A type organic compounds, introducing functional groups can modify the frontier orbitals, such as using electron‐withdrawing groups to lower the LUMO energy level, thereby significantly broadening the absorption spectrum. In this context, we propose a new molecular design strategy to develop D‐A type compounds that extend the spectral absorption range into the near‐infrared, thereby enhancing the overall efficiency of artificial photosynthetic systems.

**Scheme 1:**
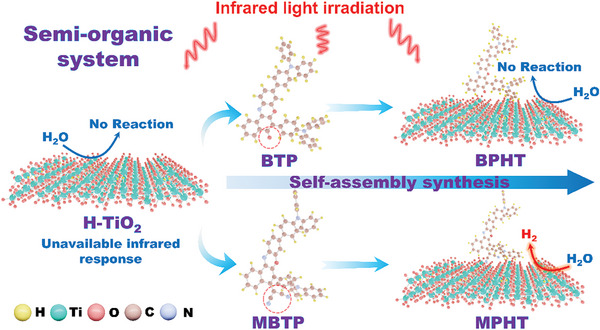
Schematic diagram of hydrogen evolution mechanism by semi‐organic artificial photosynthetic system under near‐infrared light irradiation.

We previously reported a TiO_2_ rutile/anatase twin heterophase homojunction^[^ ^20^ ^]^ and a self‐assembled structure with 2D graphitic carbon nitride nanosheets inserted between anatase TiO_2_ nanoparticles and H‐TiO_2_ nanorods.^[^ ^35^ ^]^ Although these catalyst designs demonstrated high efficiency in hydrogen evolution, they were not optimized for NIR light utilization. In this study, we introduce a new class of semi‐organic artificial photosynthetic systems that integrates molecularly engineered phenoxazinone derivatives with H‐TiO_2_ nanorods via electrostatic attraction (**Scheme** **1**). These systems form type II heterojunctions, which are well‐known for their ability to facilitate efficient charge separation and transfer, crucial for enhancing photocatalytic performance. Two specific phenoxazinone derivatives were synthesized: Bis(Triphenylamine)Phenoxazinone (BTP), which features a phenoxazinone core functionalized with two triphenylamine donor groups, enabling absorption of light up to 800 nm, and Malononitrile‐Bis(Triphenylamine)Phenoxazinone (MBTP), which includes an additional malononitrile group to further extend light absorption into the NIR region up to 1200 nm. The self‐assembly of these derivatives with H‐TiO_2_ nanorods results in the formation of semi‐organic heterojunction catalysts, denoted as BPHT (BTP/H‐TiO_2_) and MPHT (MBTP/H‐TiO_2_), respectively. Our optimized MBTP‐based system, MPHT, exhibited a remarkable photocatalytic hydrogen evolution rate of 29.4 mmol g^−1^ h^−1^ under UV–vis light irradiation. Under NIR light irradiation, MPHT achieved an impressive hydrogen evolution rate of 60.4 µmol g^−1^ h^−1^, significantly outperforming commercial rutile, P25, and pristine H‐TiO_2_. This superior performance is attributed to the tailored electronic structure of MBTP, which enhances the type II charge transfer pathway, thereby optimizing light harvesting and minimizing charge recombination losses. Advanced characterization techniques, including in situ electron spin resonance (ESR) spin‐trapping, UV–vis–NIR absorption spectroscopy, and band structure analysis, were utilized to confirm the enhanced charge separation and light absorption properties of these heterojunctions. Femtosecond transient absorption (fs‐TA) spectroscopy provided insights into the ultrafast charge dynamics, revealing efficient electron injection from the photoexcited phenoxazinone derivatives to the conduction band of H‐TiO_2_, thereby supporting the observed photocatalytic performance. Additionally, ex situ photoelectrochemical characterizations demonstrated improved charge transport properties and reduced recombination rates, underscoring the role of molecular engineering in optimizing the heterojunction's functionality. These findings present a compelling approach to advancing artificial photosynthetic systems by integrating inorganic semiconductors with strategically designed organic molecules, thereby extending the spectral range of absorption into the NIR and significantly enhancing photocatalytic hydrogen production.

## 2 Results and Discussion

### 2.1 Design and Structure Model

**Figure 1:**
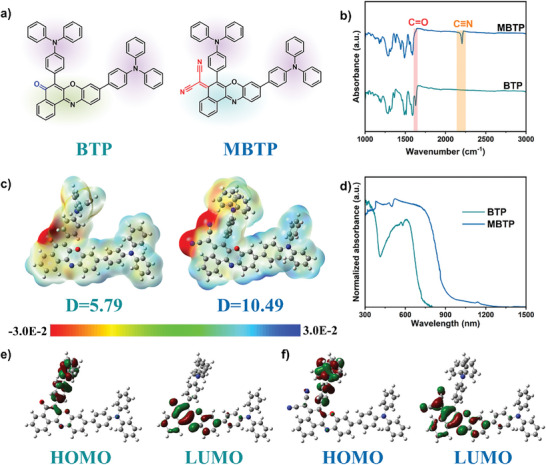
a) Theoretical modeling, b) FTIR spectra, c) electrostatic potential surface distribution, d) UV–vis–NIR absorption spectroscopy, and e,f) structure model for corresponding highest occupied molecular orbitals (HOMO) and lowest unoccupied molecular orbitals (LUMO) of BTP and MBTP, respectively.

The synthetic route for the target molecule Bis(Triphenylamine)Phenoxazinone (BTP) and its modified derivative Malononitrile‐Bis(Triphenylamine)Phenoxazinone (MBTP) is shown in **Figure** **1**a and detailed in Figure S1 (Supporting Information). BTP and MBTP were synthesized using 6,9‐dibromo‐5H‐benzo[a]phenoxazin‐5‐one/6,9‐dibromo‐5H‐benzo[a]phenoxazin‐5‐one and (4‐(diphenylamino) phenyl) boronic acid by Suzuki coupling reaction in high yields, which are further purified by column chromatography. The chemical structures are fully characterized by ^1^H nuclear magnetic resonance (NMR), Fourier transform infrared (FTIR) spectroscopies, and O 1*s* X‐ray photoelectron spectroscopy (XPS) (Figure 1b; Figures S2–S4, Supporting Information). The ^1^H‐NMR spectra of BTP and MBTP both exhibit signals corresponding to 35 hydrogen atoms (Figures S2 and S3, Supporting Information). Notably, no chemical shifts are observed in the aliphatic region, indicating the absence of hydrogen atoms on non‐aromatic structures. In contrast, the 35 aromatic hydrogen signals provide strong evidence that the chemical structures of BTP and MBTP are consistent with the designed synthetic pathways. For the O 1*s* XPS spectra in MBTP (Figure S4, Supporting Information), the peaks at 531.7 and 533.1 eV were attributed to C─O bond and adsorbed oxygen species; whereas the additional peak at 530.0 eV in the O 1*s* XPS spectra of BTP was caused by C═O bond. In the structure, we find that the 5H‐benzo[a]phenoxazin‐5‐one is a very strong acceptor, and the BTP composed by this acceptor and two triphenylamine (TPA) donors indeed displays wide absorption up to 800 nm (Figure 1d). To further extend the absorption to NIR region, we significantly increase the electron‐accepting ability by additionally attaching a malonoitrile group. As a result, the obtained target MBTP exhibits wide absorption up to 1200 nm (Figure 1d).

To deeply understand the photophysical properties of BTP and MBTP, the theoretical calculations have been systematically conducted. Compared with BTP, the electrostatic potential (ESP) distribution of MBTP displays increased concentration of reaction sites and enhanced internal electric field (Figure 1c). Meanwhile, MBTP exhibits a larger dipole moment of 10.49 D than it in BTP (5.79 D), suggesting that more free charge carriers can be easier dissociated and it can generate a stronger dipole field, thus promoting charge separation and transfer in photocatalysts. Moreover, the absorption range of BTP and MBTP is from 300–800 nm and 300–1200 nm, respectively, demonstrating that that both BTP and MBTP can well absorb the UV–vis light, even the NIR light (Figure 1d). This result further confirms that the malonoitrile group is a very simple but extremely a strong acceptor for decreasing the energy gap of molecule. Additionally, the electronic structure of phenoxazinone derivatives tightly correlates with the transportation of photoinduced charges and light absorption range. As shown in Figure 1e,f, the HOMOs of these molecules are mainly located at the donor group TPA, while the LUMOs are primarily contributed by the accepter moiety. The large separation between HOMO and LUMO well explains the red‐shifted absorptions of BTP and MBTP.

### 2.2 Morphology and Structure Characterizations

**Figure 2:**
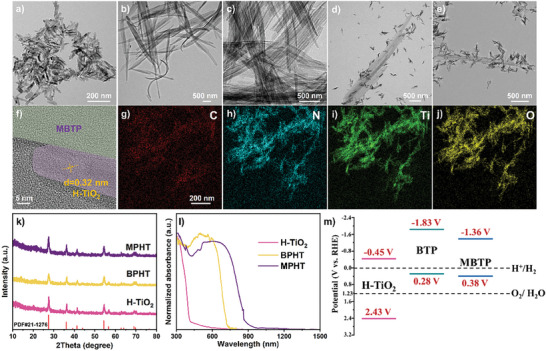
TEM images of a) H‐TiO_2_, b) BTP, c) MBTP, d) BPHT, and e) MPHT. f) HRTEM image and g–j) elemental mapping images of MPHT. k) XRD patterns, l) UV–vis–NIR absorption spectroscopy of H‐TiO_2_, BPHT, and MPHT. m) Band diagrams of H‐TiO_2_, BTP, and MBTP.

The morphologies of as‐prepared catalysts are analyzed by transmission electron microscopy (TEM). Pristine H‐TiO_2_ exhibits the nanorods structure while BTP and MBTP both show the morphology of nanobelts (**Figure** **2**a–c). After the integration of H‐TiO_2_ and BTP/MBTP, material consists of nanobelts that are decorated with nanorods (Figure 2d,e). The high‐resolution TEM image of MPHT reveals an obvious lattice fringe with a d‐spacing of 0.32 nm, ascribed to the (110) crystal planes of H‐TiO_2_ while MBTP display an amorphous structure (Figure 2f). The corresponding elemental mapping images of MPHT demonstrate the element of Oxygen (O), Titanium (Ti), Nitrogen (N), and Carbon (C) uniformly distributed in the composite catalyst (Figure 2g–j). Compared with pristine H‐TiO_2_, the basic structure of H‐TiO_2_ in as‐prepared BPHT and MPHT is well maintained as rutile phase and no any other new impurity peaks are detected (Figure 2k; Figures S5 and S6, Supporting Information). These above results confirm that two types of semi‐organic heterojunction catalysts have been successfully synthesized. As discussed below, the optimized material exhibits the excellent properties and outstanding performance of photocatalytic hydrogen evolution.

The optical properties and band position are investigated by UV–vis–NIR absorption spectroscopy and VB‐XPS spectra. Compared with pristine H‐TiO_2_, as‐prepared BPHT and MPHT are significantly redshifted into the visible region (Figure 2l). Remarkably, MPHT exhibits the redshift to NIR region, further proving a widened light absorption range. Combined with the intrinsic bandgap, valence band energy of H‐TiO_2_ and converted band energy of BTP and MBTP, the band diagrams of as‐prepared catalysts are plotted in Figure 2m and Figures S7 and S8 (Supporting Information). The detailed photocatalytic reaction mechanism will be discussed in detail combined with the following fs‐TA spectra and photoelectrochemical tests.

### 2.3 Photocatalytic Hydrogen Evolution of Catalysts

**Figure 3:**
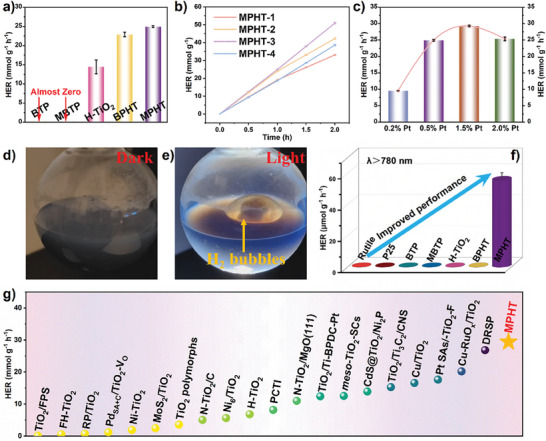
a) Photocatalytic H_2_ evolution rates over BTP, MBTP, H‐TiO_2_, BPHT, and MPHT with 0.5% Pt loading under UV–vis light (350−780 nm) irradiation. b) Time courses of photocatalytic H_2_ evolution rates toward as‐prepared MPHT‐X catalysts under UV–vis light irradiation. c) Photocatalytic H_2_ evolution rates over different loading amounts of Pt on MPHT‐3 catalyst under UV–vis light irradiation. d,e) A photograph over MPHT acquired before and under UV–vis light. f) Photocatalytic H_2_ evolution rates over Rutile, P25, BTP, MBTP, H‐TiO_2_, BPHT, and MPHT under NIR light (λ > 780 nm (780−1800 nm)) irradiation. g) Comparison of photocatalytic hydrogen evolution with previously reported TiO_2_‐based catalysts under UV–vis light irradiation.^[^ ^36^, ^37^, ^38^, ^39^, ^40^, ^41^, ^42^, ^43^, ^44^, ^45^, ^46^, ^47^, ^48^, ^49^, ^50^, ^51^, ^52^, ^53^, ^54^, ^55^ ^]^

In the context of artificial photosynthesis, we conduct the photocatalytic hydrogen evolution performance toward as‐prepared catalysts under the full spectrum light irradiation. The optimized MPHT catalyst exhibits the enhanced photocatalytic hydrogen evolution rate (HER) of 25.1 mmol g^−1^ h^−1^, which was superior to the performance of BTP, MBTP, H‐TiO_2_, and BPHT catalyst under UV–vis light irradiation (350–780 nm) (**Figure** **3**a). The mass ratio of MBTP and H‐TiO_2_ as well as the loading amount of cocatalyst Pt are optimized to achieve the excellent hydrogen evolution performance (Figure 3b,c). Excessive loading of MBTP or Pt result in the inferior hydrogen evolution rate, indicating that overloading of MBTP and cocatalyst have a detrimental effect on the hydrogen evolution process because the role of H‐TiO_2_ is almost obscured. To clearly show our excellent photocatalytic performance, we take a photograph and video of MPHT photocatalyst without and with light irradiation, respectively (Figure 3d,e; Video S1, Supporting Information). The bubbles will simultaneously produce under the condition of light irradiation while no bubbles can be found without light irradiation. This reveals that this is a light‐induced reaction and it displays an outstanding photocatalytic performance, greater than that of most previous reported TiO_2_‐based photocatalysts (Figure 3g; Table S1, Supporting Information).^[^ ^36^, ^37^, ^38^, ^39^, ^40^, ^41^, ^42^, ^43^, ^44^, ^45^, ^46^, ^47^, ^48^, ^49^, ^50^, ^51^, ^52^, ^53^, ^54^, ^55^ ^]^ Inspired by the UV–vis–NIR light absorption of MBTP and MPHT, we further investigate the corresponding photocatalytic hydrogen evolution performance under NIR light (λ>780 nm (780−1800 nm)). The results exhibit that as‐prepared MPHT catalyst owns an enhanced hydrogen evolution performance of 60.4 µmol g^−1^ h^−1^, which is much better than that of Rutile, P25, H‐TiO_2_, BTP, MBTP, and BPHT (Figure 3f). The performance trend is in well accordance with their optical absorption edge. Regarding pristine MBTP with NIR light absorption capacity, it shows a negligible performance, suggesting that it lacks photocatalytic activity on its own. Thus, we can deduce that phenoxazinone derivatives should couple with classical semiconductors and it mainly acts as a photo‐absorber. The cycling experiments and XRD patterns, XPS spectra, and TEM image of MPHT before and after reaction confirm the superior stability and well‐maintained catalyst structure (Figures S9–S11, Supporting Information). For the N 1*s*, C1*s*, Ti 2*p*, and O 1*s* XPS spectra of MPHT catalyst before and after the reaction, no significant changes were found, which also proves the stability of the catalyst structure and chemical composition during the reaction (Figures S12–S15, Supporting Information). The additional appearance of peaks of Pt 4*f* as well as the appearance of Pt nanoparticles in the TEM image proved the successful loading of Pt during the photocatalytic reaction (Figures S11 and S12f, Supporting Information).

### 2.4 Catalyst Component and Carriers’ Transportation

**Figure 4:**
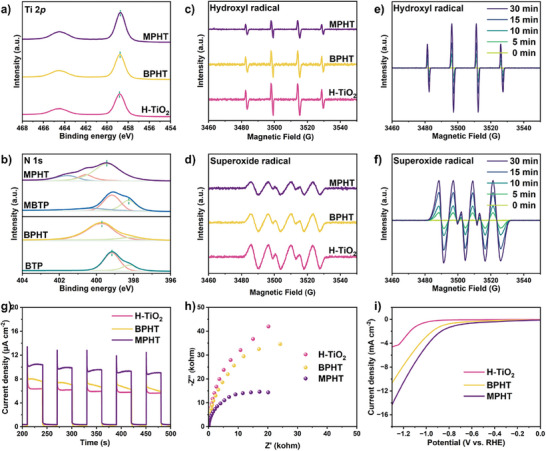
X‐ray photoelectron spectroscopy spectra of a) Ti 2*p* of H‐TiO_2_, BPHT, and MPHT and b) N 1*s* of BTP, BPHT, MBTP, and MPHT. ESR signals of H‐TiO_2_, BPHT, and MPHT c) in aqueous dispersion for DMPO‐hydroxyl radical and d) in methanol dispersion for DMPO‐superoxide radical under UV–vis–NIR light (300–2500 nm) irradiation for 5 min, respectively. In situ ESR spectra of MPHT in e) aqueous dispersion and f) methanol dispersion under dark condition (0 min) and 5, 10, 15, 30 min of UV–vis–NIR light (300–2500 nm) irradiation, respectively. g) Transient photocurrent responses, and h) EIS curves of H‐TiO_2_, BPHT, and MPHT under UV–vis light (350–780 nm) irradiation. i) LSV curves of H‐TiO_2_, BPHT, and MPHT.

XPS is tested to explore the chemical states of as‐prepared catalysts (**Figure** **4**a,b;and Figures S13–S15, Supporting Information). The ex‐situ Ti 2*p* XPS spectra in pristine H‐TiO_2_, BPHT, and MPHT show symmetrical Ti 2*p* doublets from Ti^4+^ ions while the N 1*s* XPS spectra in as‐prepared catalysts exhibits three bonds (N─C, N═C, and N≡C), respectively (Figure 4a,b). Notably, the binding energy of Ti 2*p* in BPHT and MPHT is shifted toward a lower binding energy while the binding energy of N 1*s* in BPHT and MPHT move to higher binding energy. Similar phenomenon is detected in the C 1*s* and O 1*s* spectra (Figures S14 and S15, Supporting Information). This suggests the decreased electron density around phenoxazinone derivatives and increased electron density in H‐TiO_2._ After contact each other, the photogenerated electrons will accumulate on the H‐TiO_2_ and holes will transfer from H‐TiO_2_ to phenoxazinone derivatives, following the typical Type II structure and facilitating the separation and transfer of carriers. This electron transfer is further substantiated by examining the local charge density difference (Figure S16, Supporting Information). The above results reveal the interfacial interaction between H‐TiO_2_ and BTP/MBTP, and the formation of semi‐organic junction at the interface.

Such charge transport enhancement is also confirmed by ex situ/in situ ESR spin‐trapping tests. The typical peaks of DMPO‐·O_2_^−^ and DMPO‐·OH are both detected in H‐TiO_2_, BPHT, and MPHT catalysts (Figure 4c,d). Noticeably, for the heterojunction of BPHT and MPHT, both DMPO‐·O_2_^−^ and DMPO‐·OH ESR signals are inferior than them in pristine H‐TiO_2_, proving that the reaction mechanism is a type II transfer route.^[^ ^20^ ^]^ No ESR signals of DMPO‐·O_2_^−^ and DMPO‐·OH can be detected in as‐prepared catalysts under dark condition (Figure S17, Supporting Information). Both the peak intensity of DMPO‐·O_2_^−^ and DMPO‐·OH are gradually enhanced with in situ prolonged time irradiation (Figure 4e,f). This reveals an efficient carriers’ transportation through type II structure and sufficient generation of active species. These above results offer a direct evidence of charge carrier transfer pathway under light irradiation. In order to further verify the carrier transfer mechanism of the catalyst MPHT, we have performed steady surface photovoltage spectroscopy (Figure S18, Supporting Information). The steady surface photovoltage of H‐TiO_2_ is a positive response at 300–420 nm. The photocatalyst MPHT, on the other hand, shows a signal inversion phenomenon, which implies that the electrons are enriched on the surface of H‐TiO_2_, further confirming that the flow direction of electrons is from MBTP to H‐TiO_2_, which establishes a type‐II transfer mechanism in MPHT.^[^ ^56^ ^]^

To investigate the photo‐induced charge separation efficiency of as‐prepared catalysts, transient photocurrent responses and electrochemical impedance spectra (EIS) tests are performed. As prepared MPHT catalyst has the highest photocurrent density and the photocurrent response of the catalysts shows a good reproducibility for repeated on/off cycles (Figure 4g). The addition of phenoxazinone derivatives can weaken the charge transfer resistance of H‐TiO_2_, and the as‐prepared MPHT owns a smallest arc radius (Figure 4h). This indicates that phenoxazinone derivatives coupling is beneficial to accelerating the transfer efficiency of the internal charge. The linear sweep voltammetry (LSV) curves of the catalysts, where the working overpotential of MPHT sample is significantly better than H‐TiO_2_ and BPHT (Figure 4i). More importantly, compared with BPHT, MPHT exhibits an enhanced separation and transportation efficiency, suggesting that MBTP has a more superior carrier production and mobility.

**Figure 5:**
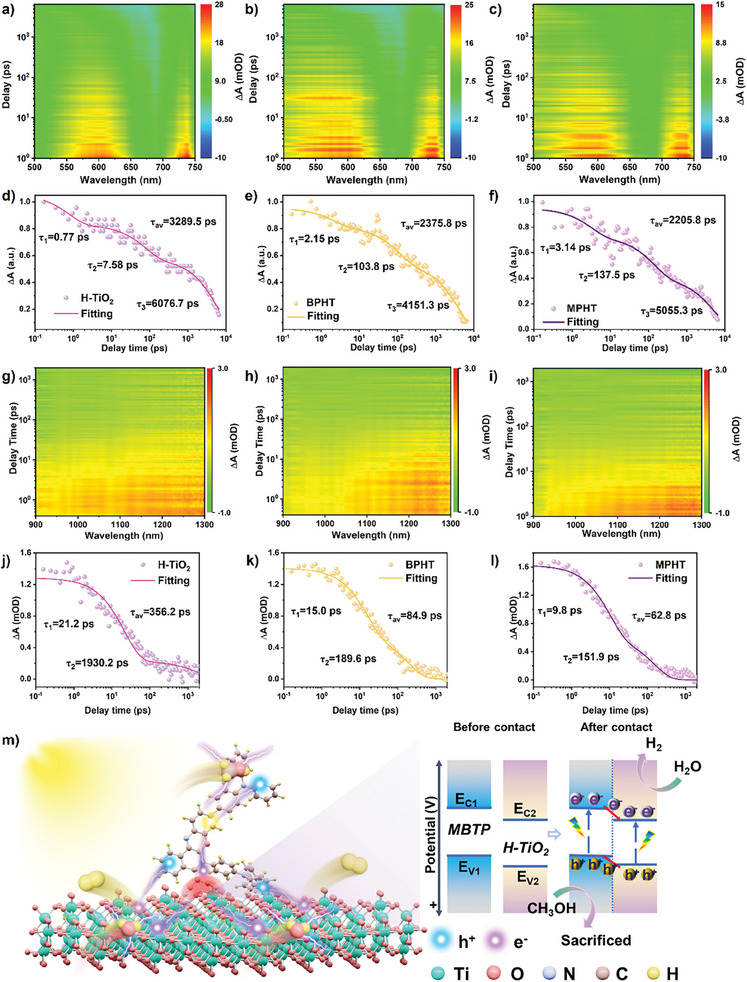
Transient absorption spectra of a) H‐TiO_2_, b) BPHT, and c) MPHT catalyst within the probe light range of 500–750 nm. Normalized transient absorption kinetics for d) H‐TiO_2_, e) BPHT, and f) MPHT catalyst after 600 nm laser excitation. Transient absorption spectra of g) H‐TiO_2_, h) BPHT, and i) MPHT catalyst within the probe light range of 900–1300 nm. Normalized transient absorption kinetics for j) H‐TiO_2_, k) BPHT, and l) MPHT catalyst after 1100 nm laser excitation. m) Illustration of carrier transfer and band structure of semi‐organic heterojunction.

To gain deeper insights into the charge separation dynamics within the semi‐organic heterojunction, we further examine the femtosecond ultrafast absorption (fs‐TA) spectra of pristine H‐TiO_2_, BPHT, and MPHT in the range of 500–750 nm as well as in the range of 900–1300 nm, respectively (**Figure** **5**a‐l; Figures S19–S24, Supporting Information). The excitation wavelengths for all catalysts are set under 400 nm, which are consistent with their band structures. Upon photoexcitation, all samples show broad and continuous absorption bands within the probe light range of 500–750 nm with a probe absorption peak of 600 nm and the probe light range of 900–1300 nm with a probe absorption peak of 1100 nm. With the probe time, a gradual decreased TA signal intensity is detected in all the as‐prepared samples, suggesting a time dependent decrease of photogenerated charges. First, the decay kinetics of these spectra were probed at 600 nm and analyzed using three exponential decays (Figure 5d–f). The calculated average carrier lifetimes (τ_av_) were found to be 3289.5 ps for H‐TiO_2_, 2375.8 ps for BPHT, and 2205.8 ps for MPHT. In addition, the decay kinetics of these spectra were probed at 1100 nm and analyzed using two exponential decays (Figure 5j–l). The calculated τ_av_ were found to be 356.2 ps for H‐TiO_2_, 84.9 ps for BPHT, and 62.8 ps for MPHT. The shorter carrier lifetime in MPHT attributed to the additional emerged channel for the interfacial charge transfer at the interface between H‐TiO_2_ and MBTP. This can promote the separation efficiency, corroborating the findings from photoelectrochemical tests.

Based on the above results and discussions, the semi‐organic heterojunction catalyst architecture for the photocatalytic hydrogen evolution is provided in Figure 5m. The enhanced performance results from the intimate interface between H‐TiO_2_ and phenoxazinone derivatives. In fact, the conduction band position of phenoxazinone derivatives is more negative than that of H‐TiO_2_ while the valence band position of H‐TiO_2_ is more positive than that of phenoxazinone derivatives. Upon light irradiation, the photogenerated electrons will spontaneously flow from phenoxazinone derivatives to H‐TiO_2_ and the holes will be transferred from H‐TiO_2_ to phenoxazinone derivatives, thus achieving the excellent separation efficiency. Moreover, due to the NIR light absorption capacity of MBTP, the optimized semi‐organic heterojunction catalyst can well absorb the broad light absorption to full utilize the photogenerated electrons for participating in the photocatalytic reaction.

## 3 Conclusion

In summary, this study focused on coupling phenoxazinone derivatives with H‐TiO_2_ and demonstrated that introducing phenoxazinone derivatives can well enhance light absorption from UV–vis to NIR light range and improve carrier transportation. Given the compatible band alignment of H‐TiO_2_ with phenoxazinone derivatives, this as‐prepared heterojunction notably boosts the separation and transfer of photogenerated carriers, resulting in the semi‐organic heterojunction catalyst achieving exceptional photocatalytic hydrogen evolution performance under UV–vis–NIR light irradiation. This research paves the way for future advancements in photocatalyst development, highlighting the potential of combining organic molecule engineering with inorganic semiconductor to enhance photocatalytic efficiency.

## Conflict of Interest

The authors declare no conflict of interest.

## Supporting information



Supporting Information

Supplemental Video 1

## Data Availability

Research data are not shared.
