# Supplementary material for: Semi‐Organic Artificial Photosynthetic System with Engineered Phenoxazinone Derivatives for Photocatalytic Hydrogen Production with Broadened Near‐Infrared Light Harvesting
Source: Adv Sci (Weinh). 2025 Feb 22;12(15):2501037. doi: 10.1002/advs.202501037 (PMC12005748; doi:10.1002/advs.202501037)
Supplement: Supplementary file 1 — Supporting Information [file ADVS-12-2501037-s001.docx]

**Supporting Information**

**Semi-Organic Artificial Photosynthetic System with Engineered Phenoxazinone Derivatives for Photocatalytic Hydrogen Production with Broadened Near-Infrared Light Harvesting**

Xiaowen Ruan,^#[1,2]^ Depeng Meng,^#[2]^ Minghua Xu,^[2]^ Guozhen Fang,^[2]^ Chunsheng Ding,^[2]^ Jing Leng,^[3]^ Xuan Wang,^[4]^ Kaikai Ba,^[5]^ Haiyan Zhang,^[2]^ Wei Zhang,^[2]^ Tengfeng Xie,^[5]^ Zhifeng Jiang,^[6]^ Jianan Dai,^*[4]^ Xiaoqiang Cui,^*[2]^ and Sai Kishore Ravi^*[1]^

[1] School of Energy and Environment, City University of Hong Kong, Tat Chee Avenue, Kowloon, Hong Kong SAR

[2] National Key Laboratory of Automotive Chassis Integration and Bionics, School of Materials Science and Engineering, Key Laboratory of Automobile Materials of MOE, Jilin Provincial International Cooperation Key Laboratory of High-Efficiency Clean Energy Materials, Electron Microscopy Center, Jilin University, 2699 Qianjin Street, Changchun 130012, China

[3] State Key Laboratory of Molecular Reaction Dynamics, Dalian Institute of Chemical Physics, Chinese Academy of Sciences, Dalian 116023, China

[4] College of Information Technology, Jilin Normal University, Siping 136000, China

[5] College of Chemistry, Jilin University, 2699 Qianjin Street, Changchun 130012, China

[6] Institute for Energy Research, Jiangsu University, Zhenjiang 212013, P. R. China

#These authors contributed equally

*Corresponding Author: Sai Kishore Ravi; Xiaoqiang Cui; Jianan Dai

E-mail: skravi@cityu.edu.hk; xqcui@jlu.edu.cn; jndai@jlnu.edu.cn

## Table of Contents

#### 1 Experimental Section S3-8.

#### 2 Results and Discussion S9-33.

#### 3 References S34-35.

**1. Experimental Section**

**Chemicals and materials.** All reagents and solvents used for the synthesis of the compound were obtained from Energy Chemical Co., Ltd and used as received without further purification. All reactions used for the synthesis of the compound were carried out using Schlenk technique under N_2_ atmosphere. Tetrabutyl (IV) titanate (Ti(OC_4_H_9_)_4_) and anhydrous ethanol (C_2_H_5_OH) were purchased from the Sinopharm Chemical Reagent Co., Ltd (China). Anhydrous methanol (CH_3_OH) was purchased from Adamas-beta (Switzerland). Trifluoromethanesulfonic acid (TfOH, CF_3_SO_3_H), commercial Rutile TiO_2_, and chloroplatinic acid hexahydrate (H_2_PtCl_6_·6H_2_O) were purchased from Sigma-Aldrich (USA). Distilled water (DI water, 18.2 MΩ cm) was employed throughout the experiments. All chemicals were analytical grade and used as received without further purification.

**Characterizations of as-prepared samples.** ^1^H NMR spectra were measured on a Zhongke-Niujin Quantum-I 400 MHz spectrometer with tetramethylsilane (TMS) as the internal standard. The absorption spectra of solids were recorded using Hitachi U-4100 UV/Vis/NIR spectrophotometer. The change of temperature was monitored by an infrared thermal imager (TESTO-869). The X-ray diffraction (XRD) patterns were recorded on a Bragg-Brentano powder diffractometer (D8-tools, Germany) using a Cu-Kα source emitting at 0.15418 nm. Fourier transform infrared (FTIR) spectra were recorded by a Nicolet iS-50 FTIR spectrometer with the KBr wafer technique. The X-ray photoelectron spectroscopy (XPS) data was collected by performing on an ESCALAB-250Xi instrument (Thermo Fisher Scientific, USA) with a monochromatic Al-Kα (1486.6 eV) radiation source and a hemisphere detector with an energy resolution of 0.1 eV. The morphologies of the samples and corresponding energy-dispersive X-ray spectroscopy (EDX) were performed on a field emission scanning electron microscopy (FESEM) (Hitachi, SU8010, Japan). A JEM-2000EX transmission electron microscope (TEM) (JEOL Co., Japan) was used to acquire the TEM images and EDX of the as-prepared samples with an acceleration voltage of 200kV. Electron spin resonance (ESR) analysis was performed using a Jeol/JES-FA200 to study the mechanism of the photocatalysts. For ESR analysis, 50 μL of DMPO (5,5-dimethyl-1-pyrroline N-oxide) with 10 mg of photocatalyst was added to 0.5 mL of distilled water/methanol.

**Synthesis of BTP.** To a mixture of 6,9-dibromo-5H-benzo[a]phenoxazin-5-one (0.400 g, 0.987 mmol), (4-(diphenylamino) phenyl) boronic acid (0.857 g, 2.960 mmol), Pd(PPh_3_)_4_ (0.058 g, 0.050 mmol) and K_2_CO_3_ (553 mg, 4.00 mmol) was added the degassed solvent mixture of toluene, ethanol, and water (V:V:V = 8:1:1, 50 mL). Then the reaction mixture was refluxed for 24 h. After cooling to room temperature, the mixture was filtered to remove inorganic salts. The filtrate was poured into water and extracted three times with CH_2_Cl_2_. The combined organic layer was washed with brine, and then dried over anhydrous MgSO_4_, and filtered. After concentration of the filtrate under reduced pressure, the resulting mixture was purified by silica gel column chromatography to afford 0.500 g (0.861 mmol, 69%) of BTP as a red solid. ^1^H NMR (400 MHz, CDCl_3_): 8.78 (d, *J* = 7.6 Hz, 1H), 8.38 (d, *J* = 7.5 Hz, 1H), 7.92 – 7.71 (m, 4H), 7.60 – 7.50 (m, 3H), 7.48 (d, *J* = 8.4 Hz, 2H), 7.42 (s, 1H), 7.29 (t, *J* = 7.8 Hz, 8H), 7.24 – 7.18 (m, 5H), 7.17 – 7.12 (m, 6H), 7.11 – 7.02 (m, 4H).

**Synthesis of MBTP.** To a mixture of 6,9-dibromo-5H-benzo[a]phenoxazin-5-one (0.400 g, 0.882 mmol), (4-(diphenylamino) phenyl) boronic acid (0.765 g, 2.650 mmol), Pd(PPh_3_)_4_ (0.058 g, 0.050 mmol) and K_2_CO_3_ (553 mg, 4.00 mmol) was added the degassed solvent mixture of toluene, ethanol, and water (V:V:V = 8:1:1, 50 mL). Then the reaction mixture was refluxed for 24 h. After cooling to room temperature, the mixture was filtered to remove inorganic salts. The filtrate was poured into water and extracted three times with CH_2_Cl_2_. The combined organic layer was washed with brine, and then dried over anhydrous MgSO_4_, and filtered. After concentration of the filtrate under reduced pressure, the resulting mixture was purified by silica gel column chromatography to afford 0.380 g (0.486 mmol, 55%) of MBTP as a red solid. ^1^H NMR (400 MHz, CDCl_3_): 8.86 (d, *J* = 6.6 Hz, 1H), 8.71 (d, *J* = 7.6 Hz, 1H), 7.82 – 7.75 (m, 1H), 7.61 – 7.49 (m, 2H), 7.35 (s, 1H), 7.35 – 7.26 (m, 4H), 7.26 – 7.18 (m, 1H), 7.18 – 7.04 (m, 7H).

**Synthesis of H-TiO_2_**. The H-TiO_2_ is synthesized by hydrothermal synthetic method ^[1]^. In detail, 10 mL of tetrabutyl (IV) titanate and 4 mL of trifluoromethanesulfonic acid were added to a polytetrafluoroethylene reactor (50 mL), and the magnetons keep stirring until well combined. Then, 10 mL of anhydrous ethanol was added to the above solution, followed by 10-min stirring. At last, the mixture in the reactor was put into a Teflon-lined stainless autoclave at 180 ℃ for 16 h. After the reaction, the resulting products were washed with DI water and anhydrous ethanol for several times, respectively, and dried in vacuum oven at 60 ℃ for 12 h.

**Synthesis of H-TiO_2_/BTP (BPHT) and H-TiO_2_/MBTP (MPHT).** The BPHT and MPHT is fabricated by a self-assembly approach via mechanical mixing at RT. Specifically, 100 mg of the as-synthesized H-TiO_2_ were dissolved in 20 mL of anhydrous ethanol to form a homogeneous solution. Then, a certain amount (0.5, 1.0, 4.0, and 9.0 mg) of BTP or MBTP was cautiously added dropwise into the above solution and stirred for several hours. After the reaction, the resulting products were washed with DI water and ethanol for several times, respectively, and dried in vacuum oven for 12 h.

**Photocatalytic H_2_ evolution experiments.** The photocatalytic H_2_ production test was performed at room temperature and atmospheric pressure. For the photocatalytic hydrogen evolution performance test under UV-vis irradiation, the steps are as follows. A closed gas circulation setup with irradiation by 300 W Xe arc lamp (Beijing Perfectlight Technology Co., Ltd) without any UV-cutoff filter (350-780 nm) was equipped. In a typical experiment, 10 mg of photocatalyst was dispersed uniformly in ca. 100 mL reaction solution in the reactor, 20% of which is CH_3_OH as the sacrificial agent. After being evacuated for 30 min, Pt as cocatalyst was deposited on the catalyst from a certain amount of H_2_PtCl_6_ aqueous solution. The reactor was always at 278 K with a circulating condensate system under stirring. The gaseous products were recorded by a GC2014C gas chromatography system with a thermal conductivity detector.

As for the testing of photocatalytic hydrogen evolution performance under near-infrared light irradiation, the procedure was as follows. A 5 mg portion of the photocatalyst was distributed in 50 mL of a methanol/H_2_O solution (1/5 methanol/H_2_O) and was sealed in a reactor. The dispersion of the photocatalyst was purged with Ar for 30 min to confirm the removal of air. After that, the sample was irradiated with near-infrared light (780-1800 nm) provided with a 780 nm cutoff filter to control the wavelength of incident light with magnetic stirring at room temperature. The gaseous products were recorded by a GC2014C gas chromatography system with a thermal conductivity detector.

**Electrochemical and photoelectrochemical measurements.** A suspension was produced by ultrasonification of 10 mg of the sample in 0.5 vol% Nafion solution-ethanol solution. The obtained suspension was evenly deposited on FTO substrates by drop coating. The acquired electrode was naturally dried in air. The electrochemical performance was conducted using a standard three-electrode system (that is work electrode: the sample, reference electrode: an Ag/AgCl and the counter electrode: a Pt foil) by using CHI650D electrochemical workstation with 0.5 M Na_2_SO_4_ electrolyte. The pH of the electrolyte is 7. Transient photocurrent responses and electrochemical impedance spectra (EIS) were conducted under a 300 W Xe lamp irradiation.

**Surface photovoltage spectroscopy Measurement.** The steady-state surface photovoltage (SPV) system consisted of a 500 W xenon lamp (CHF-XM500, Beijing Chanto Technology Co., Ltd.), a lock-in amplifier (SR830, Stanford Research Systems), and a monochromator (SBP500, Beijing Instrument Co., Ltd.). The irradiation wavelength range for SPV measurements is 300-800 nm.

**Ultrafast TA spectroscopy Measurement.** The femtosecond transient absorption setup is based on a regenerative amplified Ti: sapphire laser system from Coherent (800 nm, 35 fs, 6 mJ pulse^-1^, and 1 kHz repetition rate), nonlinear frequency mixing techniques and the Femto-TA100 spectrometer (Time-Tech Spectra LLC). Briefly, the 800 nm output pulse from the regenerative amplifier was split in two parts with a beam splitter. The transmitted part was used to pump a β-BaB_2_O_4_ crystal (BBO) which generates a 400 nm pump beam. The reflected part with less than 10% was attenuated with a neutral density filter and focused into a 2 mm thick CaF_2_ window to generate a white light continuum (WLC) from 340 nm to 750 nm or 900 nm to 1300 nm used for probe beam. The probe beam was focused with an Al parabolic reflector onto the sample. After the sample, the probe beam was collimated and then focused into a fiber-coupled spectrometer with CMOS sensors and detected at a frequency of 1 kHz. The delay between the pump and probe pulses was controlled by a motorized delay stage. The pump pulses were chopped by a synchronized chopper at 500 Hz and the induced absorbance change was calculated with two adjacent probe pulses (pump-blocked and pump-unblocked). All experiments were performed at room temperature.

**Density functional theory (DFT) calculation.** The molecular structures were optimized using DFT (S0 states) or TD-DFT (S1 states) method by means of B3LYP with the 6-31G(d, p) basis set using Gaussian 16 software package. The HOMO/LUMO distributions are calculated on the basis of optimized S0 state at the B3LYP/6-31G(d, p) level. The calculation of electrostatic potential map was performed using Gaussian 16 program at the APFD/6-311G* level of theory. All calculations were performed in the gas phase.

All spin-olarized DFT calculations were performed with the plane-ave basis set as implemented in the Vienna Ab Initio Simulation Package (VASP), and the electrons and ions interactions were described by the projector augmented wave (PAW) potential. The exchange-correlation interactions were determined by the Perdew-Burke-Ernzerhof (PBE) functional within the generalized gradient approximation (GGA). The plane wave energy cutoff of 500 eV, and the convergence criterion for the residual force and energy was set to 0.05 eV Å^−1^ and 10^−5^ eV, respectively. The empirical correction in Grimme's method (DFT+D3) was used to describe the van der Waals (vdW) interactions. The Brillouin region was sampled by the Monkhorst-Pack method with a 3 × 3 × 1 k-point mesh. A vacuum of 15Å was introduced to minimize interactions between adjacent layers in supercells.

**2. Results and Discussion**


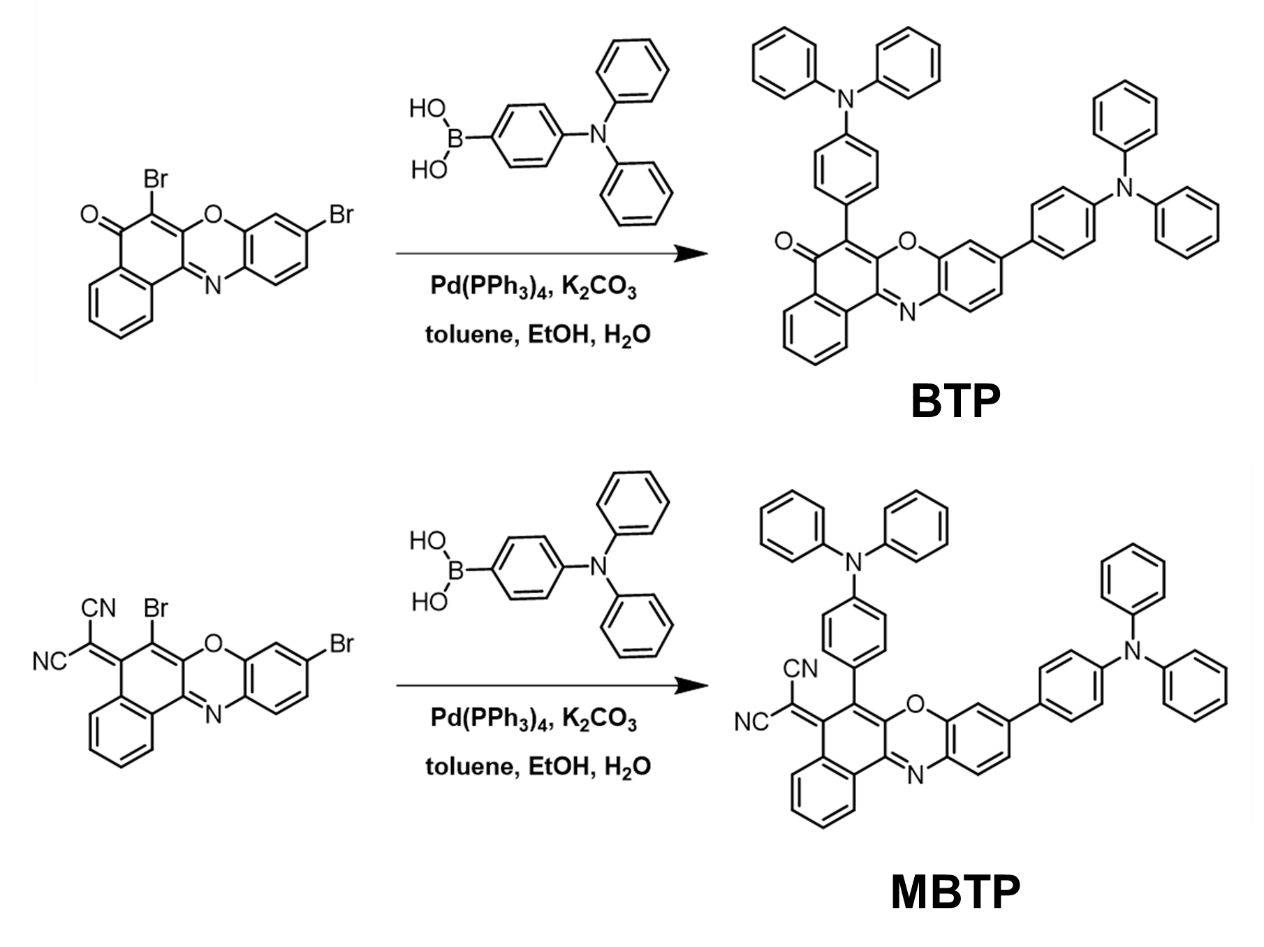


**Figure S1**. Synthetic route of BTP and MBTP.

**Figure S2**. ^1^H NMR spectrum of BTP (400 MHz, CDCl_3_).

**Figure S3**. ^1^H NMR spectrum of MBTP (400 MHz, CDCl_3_).

**

**

**Figure S4.** X-ray photoelectron spectroscopy spectra of O 1*s* of BTP and MBTP.





**Figure S5**. XRD patterns of BTP and MBTP.





**Figure S6**. XRD patterns of MPHT-X catalysts**.**





**Figure S7**. Tauc plots of H-TiO_2_**.**





**Figure S8**. VB-XPS spectra of H-TiO_2_**.**





**Figure S9**. Cycling experiments of MPHT with 1.5% Pt loading**.**





**Figure S10.** XRD patterns of as-prepared MPHT catalyst before and after reaction.


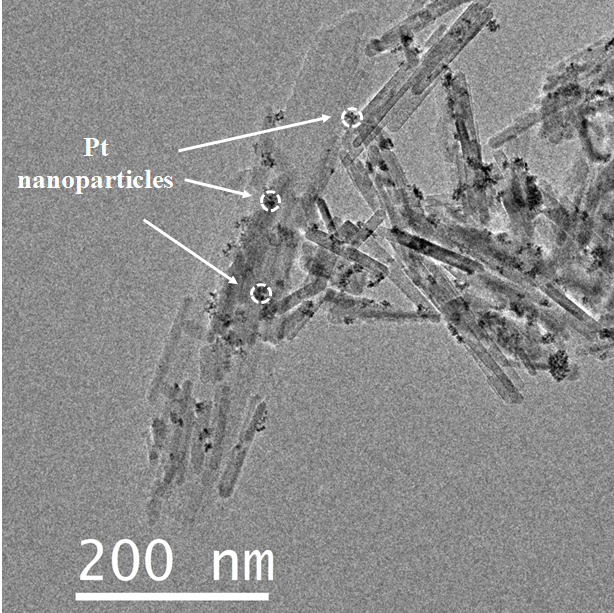


**Figure S11.** TEM image of MPHT after the photocatalytic reaction.


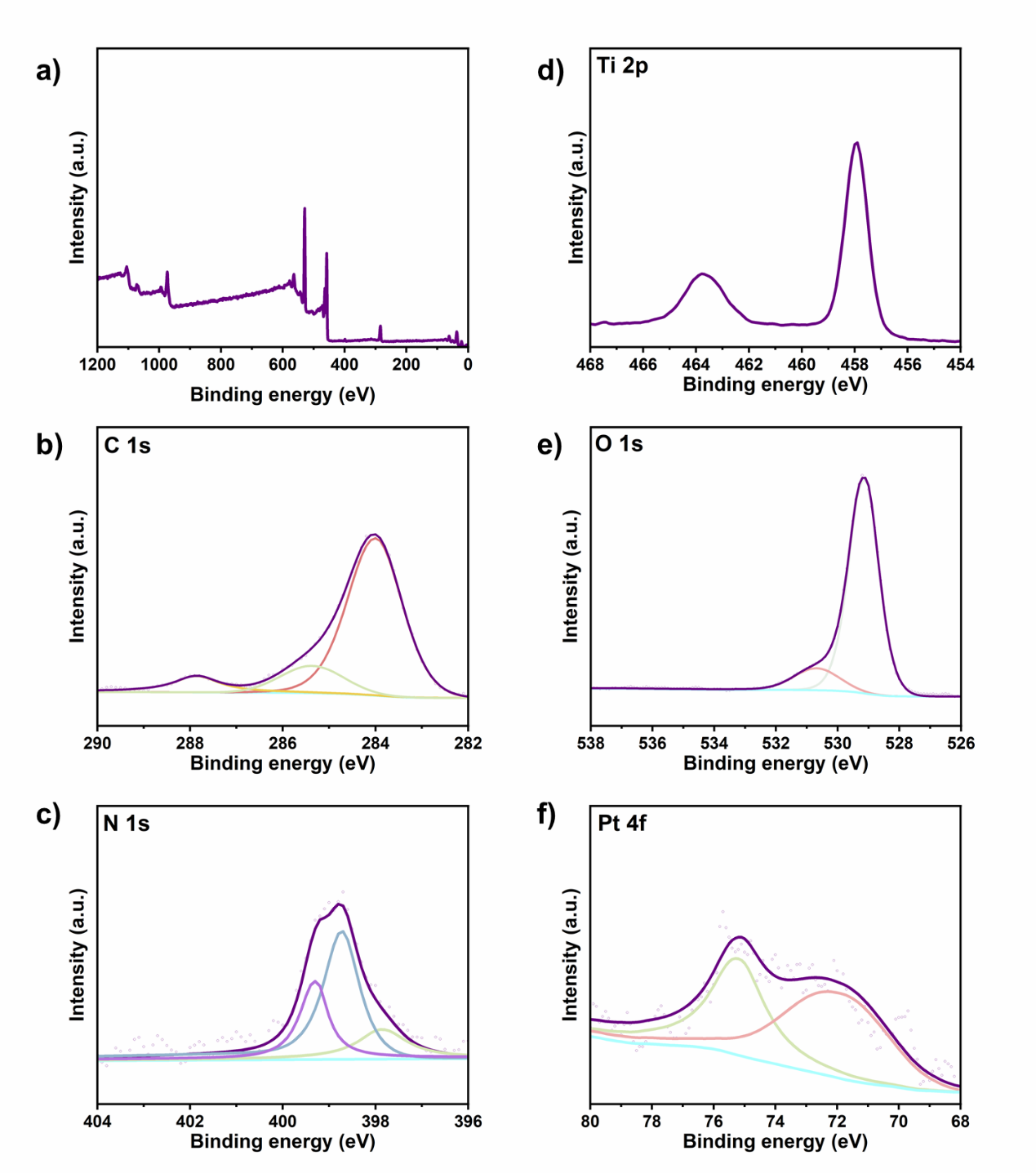


**Figure S12.** X-ray photoelectron spectroscopy spectra of all elements in MPHT after the photocatalytic reaction.





**Figure S13.** The high-resolution XPS survey spectra of H-TiO_2_, BTP, MBTP**,** BPHT, and MPHT catalyst.





**Figure S14.** X-ray photoelectron spectroscopy spectra of C 1*s* of BTP, MBTP**,** BPHT, and MPHT catalyst.

**

**

**Figure S15.** X-ray photoelectron spectroscopy spectra of O 1*s* of H-TiO_2_, BPHT, and MPHT catalyst.


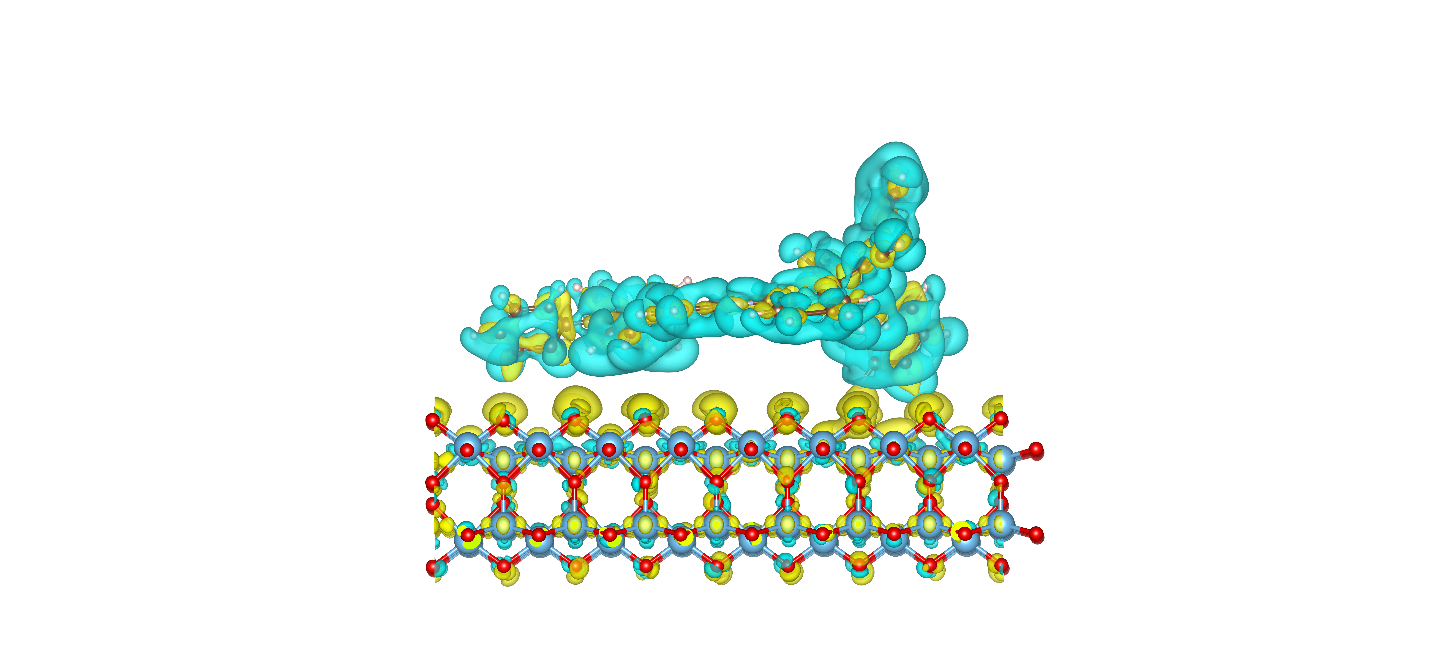


**Figure S16.** The local charge density difference of MPHT, where the light yellow and cyan areas represent electron accumulation and depletion, respectively.


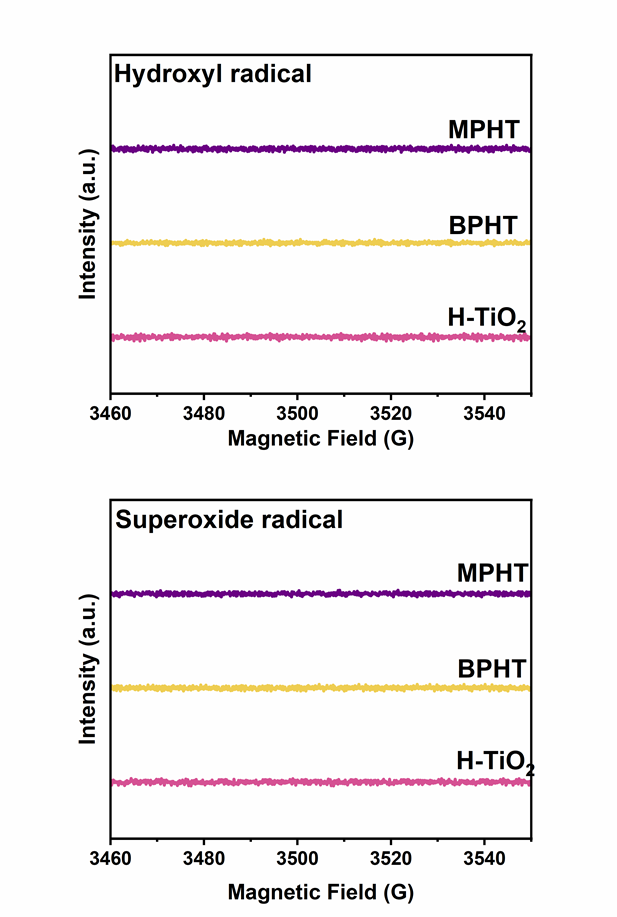


**Figure S17.** ESR signals of H-TiO_2_, BPHT, and MPHT in aqueous dispersion for DMPO-hydroxyl radical and in methanol dispersion for DMPO-superoxide radical under dark condition, respectively.





**Figure S18.** Steady surface photovoltage spectra of H-TiO_2_ and MPHT catalyst.





**Figure S19.** Transient absorption spectra of H-TiO_2_ within the probe light range of 480-750 nm.





**Figure S20.** Transient absorption spectra of BPHT within the probe light range of 480-750 nm.





**Figure S21.** Transient absorption spectra of MPHT within the probe light range of 500-750 nm.





**Figure S22.** Transient absorption spectra of H-TiO_2_ within the probe light range of 900-1300 nm.





**Figure S23.** Transient absorption spectra of BPHT within the probe light range of 900-1300 nm.





**Figure S24.** Transient absorption spectra of MPHT within the probe light range of 900-1300 nm.

**Table S1.** Summary of the hydrogen evolution of TiO_2_-based materials.

| **Materials** | **HER**  **(mmol g^-1^ h^-1^)** | **Mass (mg)** | **Year** | **Ref.** |
| --- | --- | --- | --- | --- |
| **MPHT** | **29.4** | **10** | **2024** | **This work** |
| DRSP | 26.84 | 10 | 2024 | ^[2]^ |
| Co-RuO_x_/TiO_2_ | 20.2 | 140 | 2023 | ^[3]^ |
| Pt SAs/TiO_2_–F | 17.6 | 2 | 2022 | ^[4]^ |
| Cu/TiO_2_ | 16.6 | 5 | 2019 | ^[5]^ |
| TiO_2_/Ti_3_C_2_/CNS | 15.29 | 10 | 2022 | ^[6]^ |
| CdS@TiO_2_/Ni_2_P | 13.91 | 10 | 2019 | ^[7]^ |
| meso-TiO_2_–SCs | 12.5 | 50 | 2024 | ^[8]^ |
| TiO_2_/Ti-BPDC-Pt | 12.4 | 10 | 2023 | ^[9]^ |
| N-TiO_2_/MgO(111) | 11 | 5 | 2019 | ^[10]^ |
| PCTI | 8.15 | 40 | 2023 | ^[11]^ |
| H-TiO_2_ | 6.81 | 10 | 2022 | ^[1]^ |
| Ni_6_/TiO_2_ | 5.6 | 20 | 2021 | ^[12]^ |
| N-TiO_2_/C | 5 | 20 | 2020 | ^[13]^ |
| TiO_2_ polymorphs | 3.57 | 50 | 2019 | ^[14]^ |
| MoS_2_/TiO_2_ | 2.44 | 50 | 2019 | ^[15]^ |
| Ni-TiO_2_ | 1.89 | 50 | 2020 | ^[16]^ |
| Pd_SA+C_/TiO_2_-V_O_ | 1.1736 | 10 | 2021 | ^[17]^ |
| RP/TiO_2_ | 0.681 | 20 | 2021 | ^[18]^ |
| FH-TiO_2_ | 0.566 | 50 | 2019 | ^[19]^ |
| TiO_2_/FPS | 0.0995 | 20 | 2022 | ^[20]^ |

**3 References**

[1] G. Jia, Y. Wang, X. Cui, H. Zhang, J. Zhao, L. H. Li, L. Gu, Q. Zhang, L. Zheng, J. Wu, Q. Wu, D. J. Singh, W. Li, L. Zhang, W. Zheng, *Matter* **2022**, 5, 206.

[2] X. Ruan, D. Meng, C. Huang, M. Xu, D. Jiao, H. Cheng, Y. Cui, Z. Li, K. Ba, T. Xie, L. Zhang, W. Zhang, J. Leng, S. Jin, S. K. Ravi, Z. Jiang, W. Zheng, X. Cui, J. Yu, *Adv. Mater.* **2024**, 36, 2309199.

[3] J. Shen, C. Luo, S. Qiao, Y. Chen, K. Fu, J. Xu, J. Pei, Y. Tang, X. Zhang, H. Tang, H. Zhang, C. Liu, *Adv. Funct. Mater.* **2024**, 34, 2309056.

[4] S.-M. Wu, I. Hwang, B. Osuagwu, J. Will, Z. Wu, B. B. Sarma, F.-F. Pu, L.-Y. Wang, Z. Badura, G. Zoppellaro, E. Spiecker, P. Schmuki, *ACS Catal.* **2023**, 13, 33.

[5] B.-H. Lee, S. Park, M. Kim, A. K. Sinha, S. C. Lee, E. Jung, W. J. Chang, K.-S. Lee, J. H. Kim, S.-P. Cho, H. Kim, K. T. Nam, T. Hyeon, *Nat. Mater.* **2019**, 18, 620.

[6] H. Zeng, Z. Li, G. Li, X. Cui, M. Jin, T. Xie, L. Liu, M. Jiang, X. Zhong, Y. Zhang, H. Zhang, K. Ba, Z. Yan, Y. Wang, S. Song, K. Huang, S. Feng, *Adv. Energy Mater.* **2022**, 12, 2102765.

[7] K. Wu, P. Wu, J. Zhu, C. Liu, X. Dong, J. Wu, G. Meng, K. Xu, J. Hou, Z. Liu, X. Guo, *Chem. Eng. J.* **2019**, 360, 221.

[8] S. Yin, L. Liu, J. Li, H. Wu, Z. Lv, Y. He, J.-Y. Zhang, P. Zhang, Z. Zhao, D. Zhao, K. Lan, *J. Am. Chem. Soc.* **2024**, 146, 1701.

[9] X. He, Y. Ding, Z. Huang, M. Liu, M. Chi, Z. Wu, C. U. Segre, C. Song, X. Wang, X. Guo, *Angew. Chem. Int. Ed.* **2023**, 62, e202217439.

[10] Y. Li, Y.-K. Peng, L. Hu, J. Zheng, D. Prabhakaran, S. Wu, T. J. Puchtler, M. Li, K.-Y. Wong, R. A. Taylor, S. C. E. Tsang, *Nat. Commun.* **2019**, 10, 4421.

[11] H. S. Moon, K.-C. Hsiao, M.-C. Wu, Y. Yun, Y.-J. Hsu, K. Yong, *Adv. Mater.* **2023**, 35, 2200172.

[12] F. Tian, J. Chen, F. Chen, Y. Liu, Y. Xu, R. Chen, *Appl. Catal. B* **2021**, 292, 120158.

[13] B. Yan, D. Liu, X. Feng, M. Shao, Y. Zhang, *Adv. Funct. Mater.* **2020**, 30, 2003007.

[14] H. Xiong, L. Wu, Y. Liu, T. Gao, K. Li, Y. Long, R. Zhang, L. Zhang, Z.-A. Qiao, Q. Huo, X. Ge, S. Song, H. Zhang, *Adv. Energy Mater.* **2019**, 9, 1901634.

[15] W. Wang, S. Zhu, Y. Cao, Y. Tao, X. Li, D. Pan, D. L. Phillips, D. Zhang, M. Chen, G. Li, H. Li, *Adv. Funct. Mater.* **2019**, 29, 1901958.

[16] M. Xiao, L. Zhang, B. Luo, M. Lyu, Z. Wang, H. Huang, S. Wang, A. Du, L. Wang, *Angew. Chem. Int. Ed.* **2020**, 59, 7230.

[17] T. Wang, X. Tao, X. Li, K. Zhang, S. Liu, B. Li, *Small* **2021**, 17, 2006255.

[18] S. Li, Y. H. Ng, R. Zhu, S. Lv, C. Wu, Y. Liu, L. Jing, J. Deng, H. Dai, *Appl. Catal. B* **2021**, 297, 120412.

[19] C. Gao, T. Wei, Y. Zhang, X. Song, Y. Huan, H. Liu, M. Zhao, J. Yu, X. Chen, *Adv. Mater.* **2019**, 31, 1806596.

[20] B. Xia, B. He, J. Zhang, L. Li, Y. Zhang, J. Yu, J. Ran, S.-Z. Qiao, *Adv. Energy Mater.* **2022**, 12, 2201449.
